# Case Report: Acute Amebic Colitis Triggered by Colonoscopy: Exacerbation of Asymptomatic Chronic Infection with *Entamoeba histolytica* Accompanied by Dysbiosis

**DOI:** 10.4269/ajtmh.19-0396

**Published:** 2019-10-07

**Authors:** Yasuaki Yanagawa, Takahiro Arisaka, Satoru Kawai, Kumiko Nakada-Tsukui, Atsuhito Fukushima, Hideyuki Hiraishi, Yuichi Chigusa, Hiroyuki Gatanaga, Shinichi Oka, Tomoyoshi Nozaki, Koji Watanabe

**Affiliations:** 1 AIDS Clinical Center, National Center for Global Health and Medicine, Tokyo, Japan;; 2 Joint Research Center for Human Retrovirus Infection, Kumamoto University, Kumamoto, Japan;; 3 Department of Gastroenterology, Dokkyo Medical University, Tochigi, Japan;; 4 Department of Tropical Medicine and Parasitology, Dokkyo Medical University, Tochigi, Japan;; 5 Department of Parasitology, National Institutes of Infectious Diseases, Tokyo, Japan;; 6 Department of Infection Control and Clinical Laboratory Medicine, Dokkyo Medical University, Tochigi, Japan;; 7 Graduate School of Medicine, The University of Tokyo, Tokyo, Japan

## Abstract

Recent data show that the gut microbiome plays a role in determining the clinical outcome of *Entamoeba histolytica* infection. We report the case of a patient who developed recurrent acute amebic colitis (second episode of acute colitis) after colonoscopy. Genotyping of *E. histolytica* revealed that she developed a second episode of acute amebic colitis with the same genotype as that of the first episode, indicating chronic infection had persisted asymptomatically for > 10 months between the first and second episodes. Analysis of the gut microbiome, in addition to the clinical findings, suggested that dysbiosis at colonoscopy induced the change in the clinical form of *E. histolytica* infection from asymptomatic chronic infection to symptomatic colitis.

## CASE DESCRIPTION

We present the case of a previously healthy, HIV-1–negative, 41-year-old female commercial sex worker who developed frequent watery diarrhea (8 times/day) after colonoscopy, performed as part of a 10-month follow-up after metronidazole monotherapy for amebic colitis.

Twenty months before presentation, the patient had watery diarrhea (> 10 times/day). Although the frequency improved to twice daily following treatment with probiotics (LAC-B^®^ tablets, Kowa Company, Ltd., Japan), chronic diarrhea with loose stool continued. Probiotics were continued without further examination. Thereafter, 10 months before the current presentation, colonoscopy was performed as part of examination for chronic diarrhea. The patient was diagnosed with amebic colitis based on 1) identification of *Entamoeba* in multiple biopsy specimens from colonic circular mucosal aphthous ulcers with mucosal edema and erythema in the cecum and 2) positive results of polymerase chain reaction (PCR) for *Entamoeba histolytica* in loose stool (first diagnosis of amebic colitis). Her diarrhea improved to normal stool after treatment with oral metronidazole (1,500 mg/day for 10 days). At 1 and 2 months after metronidazole treatment, examination of formed stool samples for *E. histolytica* with stool ova and parasite and PCR were negative. Although we had recommended follow-up colonoscopy to check for mucosal lesions, the patient declined because she had completely recovered from abdominal symptoms. No luminal agents were used at that time to eradicate the parasite.

**Figure 1.**
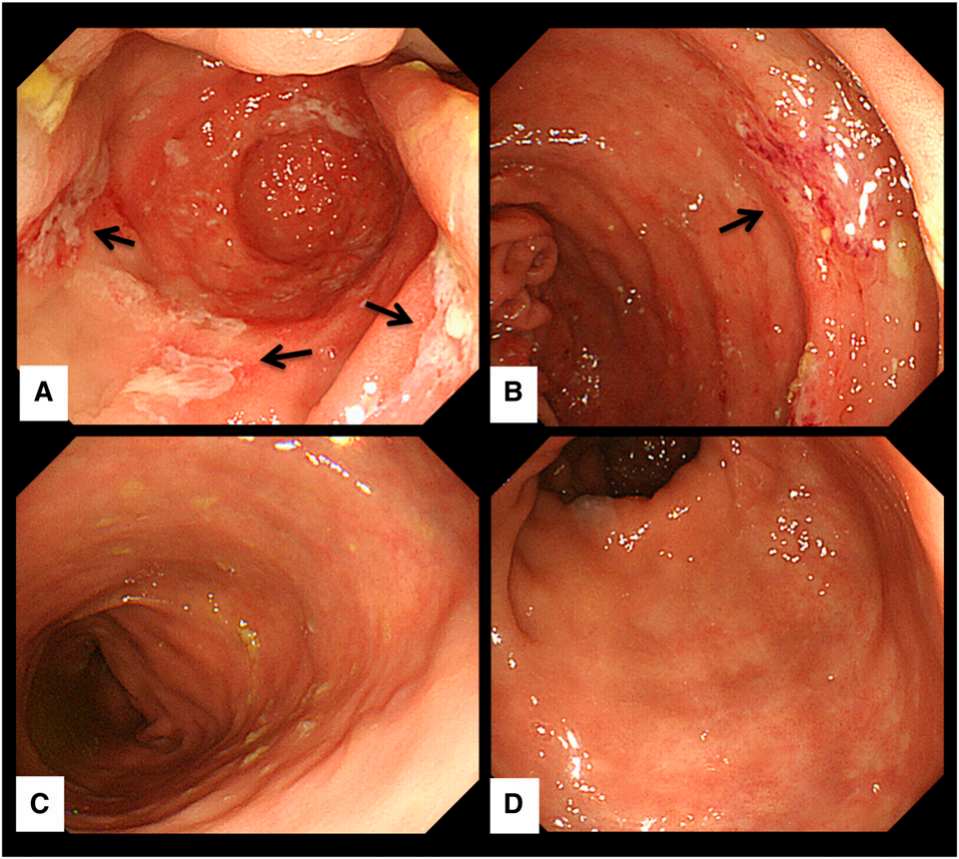
Colonoscopy images during asymptomatic chronic infection with *Entamoeba histolytica*. Multiple erosions with exudates surrounded by edematous mucosa were identified in the cecum and ascending colon (black arrows). Other lesions of the large intestine were intact. (**A**): Cecum, (**B**): ascending colon, (**C**): descending colon, and (**D**): rectum.

Ten months later, the patient finally agreed to undergo follow-up colonoscopy. Multiple ulcerative mucosal lesions were identified in the cecum (Figure 1), although the patient was completely asymptomatic. Furthermore, several days after colonoscopy, she developed frequent watery diarrhea, which continued for > 2 weeks. Polymerase chain reaction testing of diarrheal stool was positive for *E. histolytica*, although *Entamoeba* was not identified in biopsy specimens sampled on colonoscopy (second diagnosis of amebic colitis). Examination of the genotypes of *E. histolytica*, based on DNA sequences targeting short tandem repeats on transfer RNA genes (tRNA STR) in stool samples, showed the same genotype at the first and second diagnoses (Supplemental Figure 1). Metronidazole (1,500 mg/day for 10 days) was reinitiated with the diagnosis of recurrent amebic colitis after long-term chronic subclinical infection. Paromomycin (1,500 mg/day for 7 days) was administered for luminal treatment 1 month after metronidazole treatment. Finally, colonoscopy at 4 months after paromomycin treatment confirmed that all colonic ulcerative lesions had healed.

**Figure 2.**
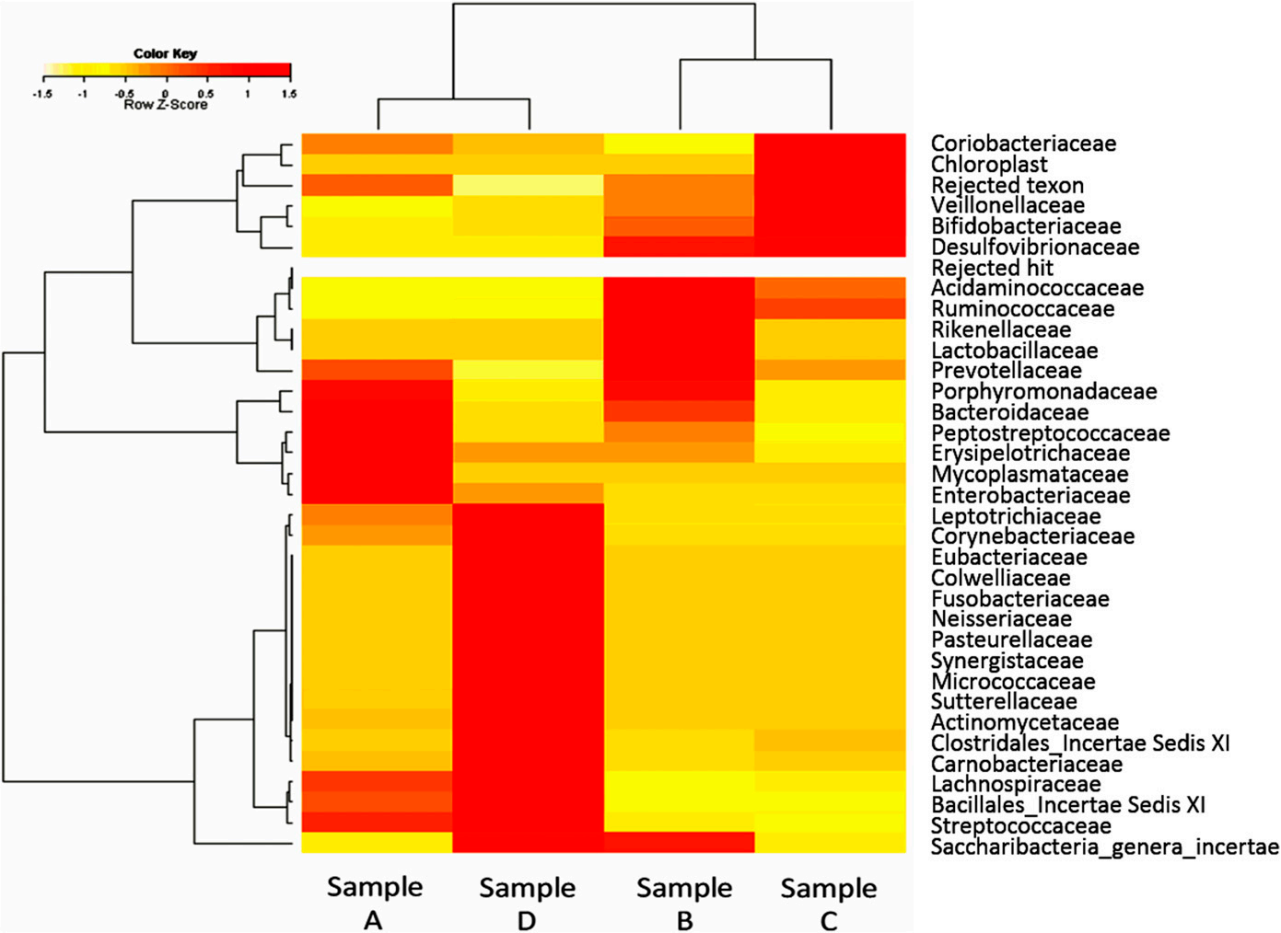
Gut microbiome composition by family in stool samples collected at four different time points during the clinical course of the patient. Clustering (correlation-average) family. Mean taxonomic levels of four bacterial families (*Prevotellaceae*, *Ruminococcaceae*, *Bifidobacteriaceae*, and *Veillonellaceae*) were higher in the AS group (samples B and C) than the DS group (samples A and D), whereas those of 12 bacterial families (*Lachnospiraceae*, *Streptococcaceae*, *Erysipelotrichaceae*, *Bacteroidaceae*, *Micrococcaceae*, *Peptostreptococcaceae*, *Actinomycetaceae*, *Carnobacteriaceae*, *Enterobacteriaceae*, *Bacillales Family XI Incertae Sedis*, *Corynebacteriaceae*, and *Leptotrichiaceae*) were higher in the DS group (samples A and D). *Descriptions of each sample. Sample A: stool sample obtained during symptomatic amebic colitis (watery stool collected 4 weeks after colonoscopy). Sample B: stool sample obtained during chronic infection, formed stool at 1 month after metronidazole treatment (without paromomycin). Sample C: stool sample obtained in the absence of *Entamoeba histolytica* infection, formed stool at 8 months after paromomycin treatment (2 months after bowel cleansing). Sample D: Intestinal fluid sample obtained just after bowel cleansing in the absence of *E. histolytica* infection at 6 months after paromomycin treatment.

To assess the impact of the gut microbiome on clinical outcome of *E. histolytica* infection, we collected stool samples at four planned time points during and after treatment of the second episode of amebic colitis, after obtaining informed consent from the patient. We collected the following: Sample A, watery stool at the time of amebic colitis before metronidazole; Sample B, formed stool after metronidazole therapy but before paromomycin; Sample C, formed stool collected long-term after paromomycin and colonoscopy; and Sample D, stool after bowel cleansing for colonoscopy (Supplemental Figure 2). We analyzed the microbiome composition with a universal primer based on the V3-V4 hypervariable region of prokaryotic 16S rDNA using an Illumina MiSeq next-generation sequencer. The gut microbiome of Sample A was similar to that of Sample D, which was quite different from both Samples B and C (Supplemental Figure 3). The microbiome composition of Samples B and C represented that during asymptomatic infection with *E. histolytica* (AS group), whereas the composition of Samples A and D were considered the same as those during a more invasive *E. histolytica* infection (DS group). Next, to identify the microbiome composition responsible for the clinical form of *E. histolytica* infection, we compared the microbiome composition in the AS group with that in the DS group in the presence or absence of *E. histolytica* (comparison 1, Sample B versus A and comparison 2, Sample C versus D). The mean taxonomic levels of four bacterial families (*Prevotellaceae*, *Ruminococcaceae*, *Bifidobacteriaceae*, and *Veillonellaceae*) were significantly higher in the AS group (Samples B and C) than in the DS group (Samples A and D), whereas those of 12 bacterial families (*Lachnospiraceae*, *Streptococcaceae*, *Erysipelotrichaceae*, *Bacteroidaceae*, *Micrococcaceae*, *Peptostreptococcaceae*, *Actinomycetaceae*, *Carnobacteriaceae*, *Enterobacteriaceae*, *Bacillales Family XI Incertae Sedis*, *Corynebacteriaceae*, and *Leptotrichiaceae*) were significantly higher in the DS group (Samples A and D) (Figure 2, Supplemental Figure 4).

## DISCUSSION

Amebiasis, a protozoan infection caused by *E. histolytica*, is endemic in developing countries and is a leading cause of severe diarrhea and death worldwide.^1^ It has also been reported that amebiasis is a spreading sexually transmitted infection in East Asian developed countries^2,3^; however, it is regarded as a neglected disease in Japan. No luminal agents have been available in Japan until recently, with paromomycin approved in 2013. In addition, there is no clear evidence showing a preventive effect of luminal agents against recurrence of invasive amebiasis in people at high risk for reinfection. One study showed that the recurrence rate in 6 years of follow-up was about 13% with or without luminal treatment in the HIV-1 cohort; however, this finding cannot be applied to HIV-1–negative patients or to the present case.^4^ Such circumstances cause the unfavorable situation that paromomycin is not routinely administered after treatment with metronidazole for invasive amebiasis in Japan.

The common clinical form of amebiasis is mild, self-limiting colitis, with less frequent invasive disease.^5^ Although determining factors of the clinical form of *E. histolytica* infection are unclear, recent human and animal data indicate that the gut microbiota and host and/or pathogen genetic factors play important roles.^6–8^ It is also well known that bowel cleansing induces disturbance of the normal gut flora (dysbiosis).^9^ In the present case, we confirmed that the genotypes of *E. histolytica* in the first and second episodes of colitis were identical using a genotyping method targeting six different tRNA STR loci (genotype J13) (Supplemental Figure 1). In addition, the colonic ulcers seen on colonoscopy were completely healed after luminal therapy. From these results, we determined that the cecal ulcers seen in the follow-up colonoscopy were caused by asymptomatic chronic infection with *E. histolytica* that had persisted for > 10 months after metronidazole monotherapy, following the first episode of amebic colitis. Thereafter, the patient developed a second episode of amebic colitis because of genetically identical *E. histolytica* with frequent watery diarrhea after follow-up colonoscopy. It is possible that dysbiosis caused by bowel preparation for colonoscopy triggered the second episode.

From analysis of the gut microbiome at different time points, we identified four and 12 bacterial families in the asymptomatic state and in the diarrheal state, respectively. Among these, *Bifidobacteriaceae*, which was higher in the AS group, is reported to be associated with the severity of amebiasis according to previous reports. One cross-sectional study from India reported that the *Bifidobacteriaceae* burden was higher in *E. histolytica* cyst–containing stool samples than in samples from uninfected individuals.^10^ Another cross-sectional study from this research group showed that *Bifidobacteriaceae* was reduced in patients with amebic liver abscess as compared with asymptomatically infected individuals.^11^ Probiotics containing *Bifidobacteriaceae* are reported to be effective in infectious colitis.^12,13^ Moreover, in the present case, treatment with probiotics containing *Bifidobacteriaceae* (LAC-B tablets) was given before the first diagnosis of amebiasis, which had a partial effect on diarrhea caused by amebic colitis but failed to eradicate this protozoan. These results indicate that colonization of *Bifidobacteriaceae* appears to suppress gut inflammation owing to *E. histolytica* infection.

There are several limitations to be considered in this study. First, one case report is insufficient to reach a conclusion regarding effects of the gut microbiome on disease severity in *E. histolytica* infection. Further data from in vivo and in vitro studies are needed. Second, it remains possible that the present patient acquired the second infection from the same source.^14^ In addition, the negative results of stool PCR tests for *E. histolytica* after metronidazole monotherapy may indicate reinfection with *E. histolytica* because of the considerably high but imperfect sensitivity of PCR in clinical settings.^15^ Third, the potential effects of probiotics and paromomycin on the gut microbiome must be taken into account, although we waited for a long interval before collecting samples, to minimize these effects. Finally, the mechanical effects of disruption of the protective mucus barrier by colonoscopy could be a plausible explanation for the exacerbation of *E. histolytica* infection.^16^

In conclusion, this is the first report of a genetically identical enteric pathogen causing recurrent colitis in the same patient after > 10 months of asymptomatic colonization, probably because of dysbiosis induced by bowel cleansing before colonoscopy.

## Supplemental figures

Supplemental materials
