# Supplementary material for: Case Report: Acute Amebic Colitis Triggered by Colonoscopy: Exacerbation of Asymptomatic Chronic Infection with Entamoeba histolytica Accompanied by Dysbiosis
Source: Am J Trop Med Hyg. 2019 Oct 7;101(6):1384–7. doi: 10.4269/ajtmh.19-0396 (PMC6896890; doi:10.4269/ajtmh.19-0396)
Supplement: Supplementary file 1 [file tpmd190396.SD1.pdf]

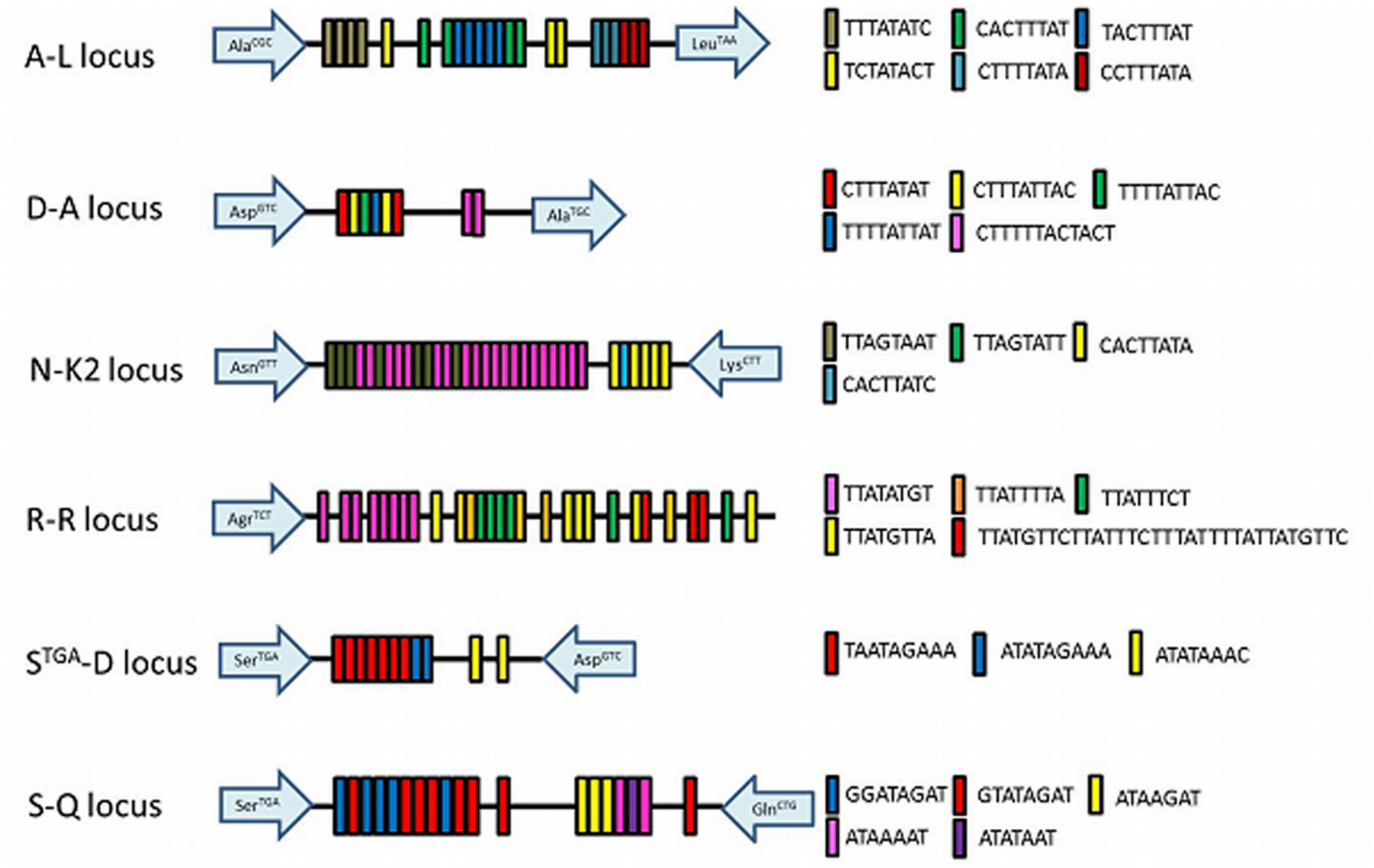

Supplementary Figure 1. Sequence patterns of 6 transfer RNA loci of *Entamoeba histolytica*. Genotyping was determined by the patterns of short tandem repeats in tRNA. *E. histolytica* DNA sequences extracted from stool samples at first and second episode of diarrhea were completely matched in 6 different loci (subtyping of each loci, J8AL, 15DA, J3NK, 5RR, 9SD, and J1SQ).

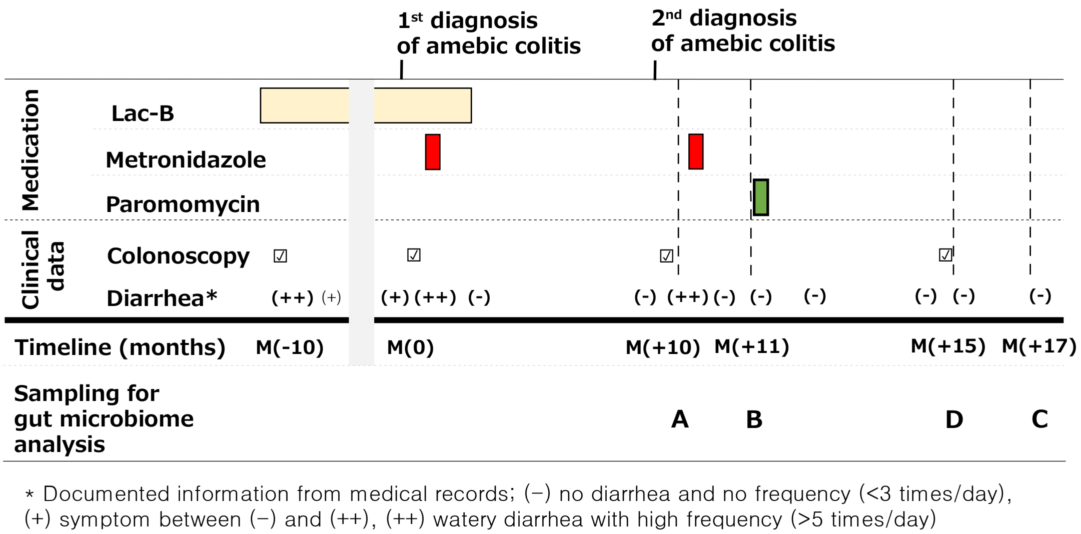

Supplementary Figure 2. Timeline of the clinical course and time points of collecting samples.

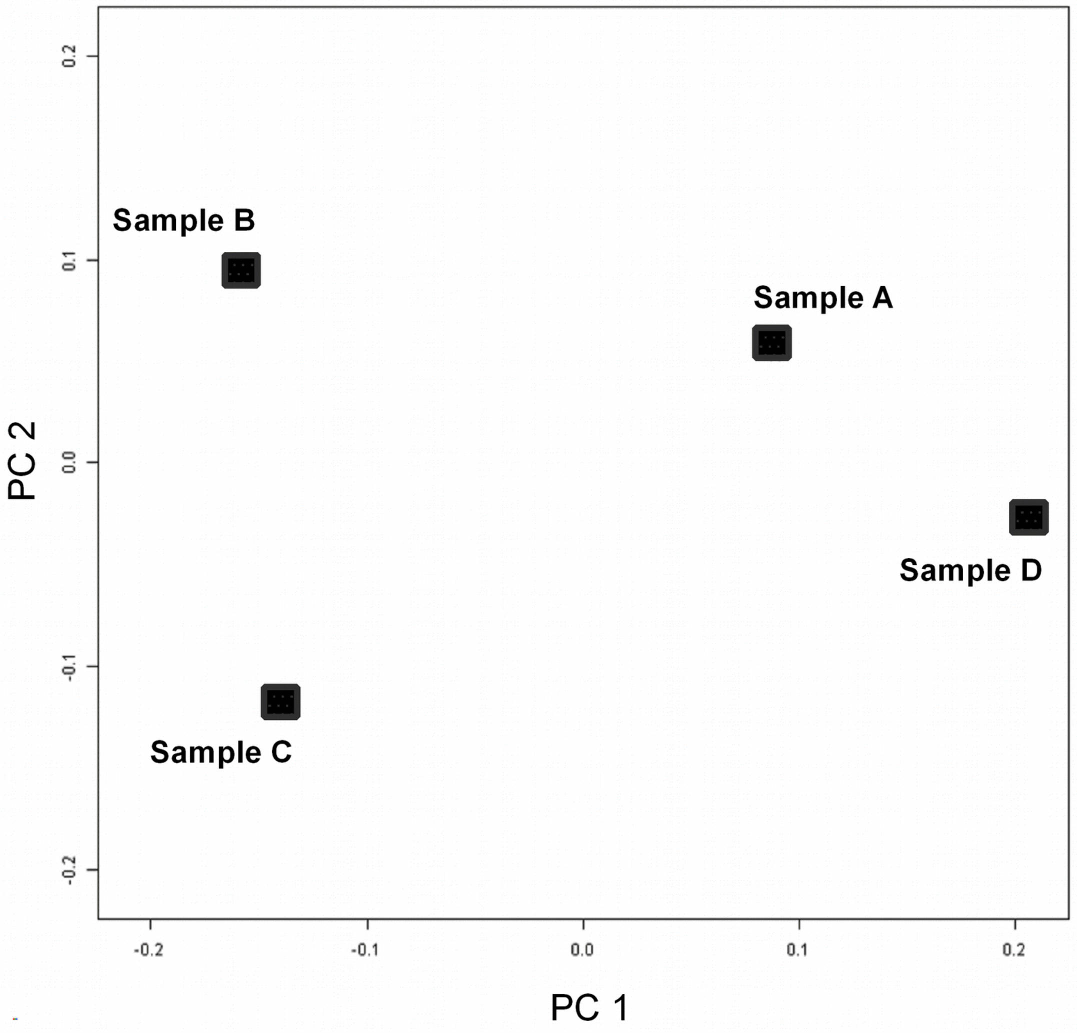

Supplementary Figure 3. Gut microbiome composition by family of stool samples collected at four different time points during clinical course of the patient. Principal Component Analysis Family. The gut microbiome of sample A was similar to that of sample D (samples during invasive diseases: DS group), which was quite different from both sample B and C (samples during asymptomatic infection: AS group). * Descriptions of each sample: Sample A: stool sample obtained during symptomatic amebic colitis (watery stool collected 4 weeks after colonoscopy). Sample B: stool sample obtained during chronic infection, formed stool at 1 month after metronidazole treatment (without paromomycin). Sample C: stool sample obtained in the absence of *E. histolytica* infection, formed stool at 8 months after paromomycin treatment (2 months after bowel cleansing). Sample D: Intestinal fluid sample obtained just after bowel cleansing in the absence of *E. histolytica* at 6 months after paromomycin treatment.

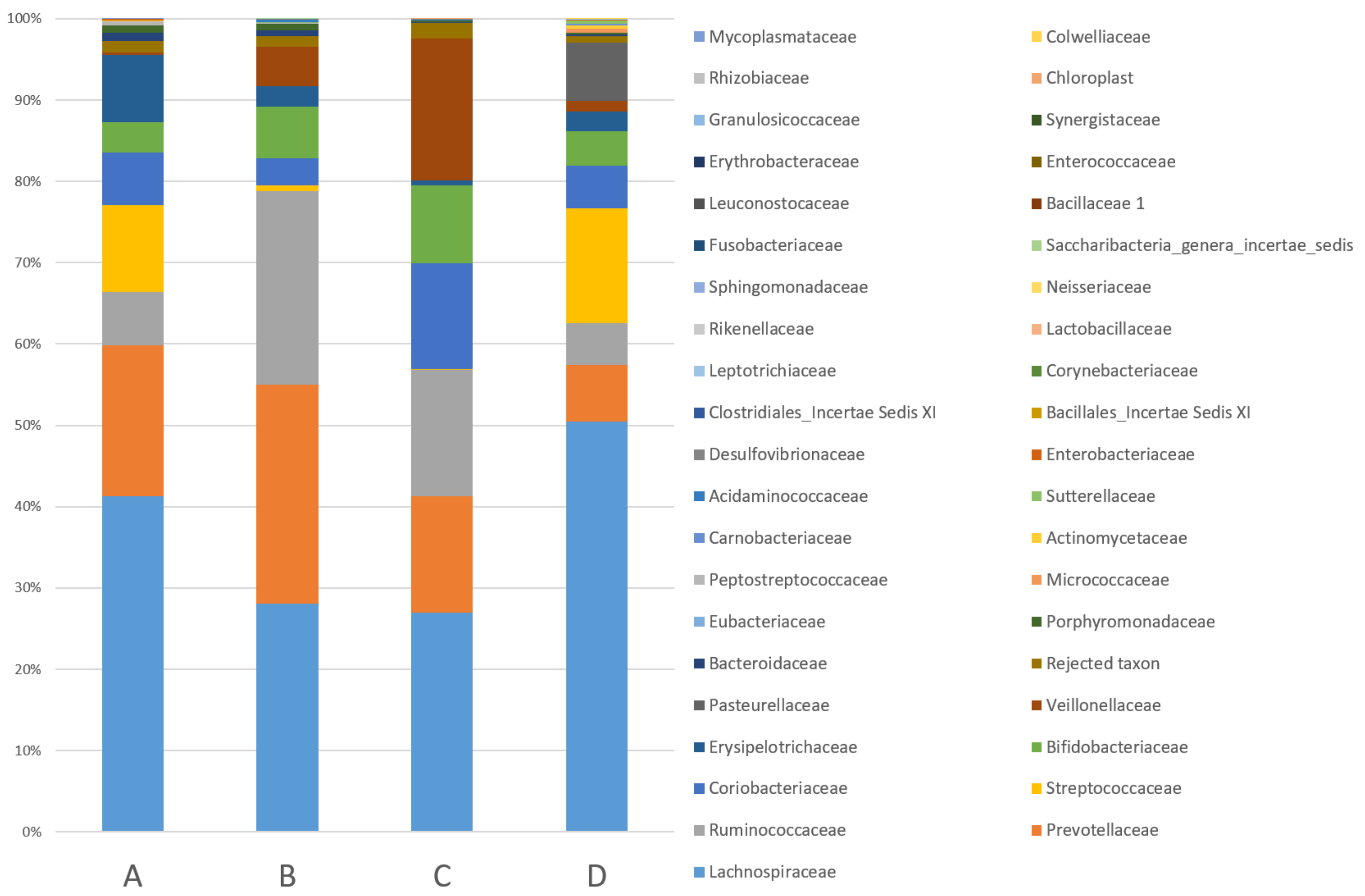

Supplementary Figure 4. Gut microbiome analysis results by family level of stool. The bar charts show the taxonomic profiles obtained for each the four stool samples. The V3-V4 region of 16S rDNA was amplified using 341f/R806 for Bacteria primer set. Sequencing was conducted using a paired-end, 2x280-bp cycle run on an Illumina MiSeq sequencing system. Analyses of sequencing reads were performed manually using the Ribosomal Database Project Multiclassifier tool.
